# Choroidal thickness changes measured by enhanced depth imaging optical coherence tomography in third trimester pregnant women

**DOI:** 10.1186/s40942-018-0118-y

**Published:** 2018-04-23

**Authors:** Lorenzo Ferro Desideri, Fabio Barra, Simone Ferrero, Aldo Vagge

**Affiliations:** 1 Department of Ophthalmology, Ospedale Policlinico San Martino, Largo R. Benzi 10, 16131 Genoa, Italy; 2 Academic Unit of Obstetrics and Gynecology, Ospedale Policlinico San Martino, Largo R. Benzi 10, 16131 Genoa, Italy; 3 Department of Neurosciences, Rehabilitation, Ophthalmology, Genetics, Maternal and Child Health (DiNOGMI), University of Genoa, Genoa, Italy

**Keywords:** Choroid, Choroidal thickness, Pregnancy, Third trimester, Optical coherence tomography, Hormonal changes

## Abstract

The aim of this article is to underline the effect of pregnancy on the variations of choroidal thickness caused by hormonal and haemodynamic changes.

Dear editor,

We read with great interest the article “Comparative analysis of choroidal thickness in third trimester pregnant women” recently published in the International Journal of Vitreous and Retina.

In this cross-sectional study Benfica et al. [1] analyzed the measurements of choroidal thickness in healthy third trimester pregnant women in comparison with healthy non-pregnant women using optical coherence tomography (OCT), reporting no statistical difference both in the mean subfoveal choroidal thickness and in the ten different measurements of choroidal thickness in the macula [1]. The authors should be congratulated for the prospective study design, the adoption of the enhanced depth imaging OCT (EDI-OCT) technique, which allows a better visualization of choroidal morphology compared with the traditional spectral-domain OCT (SD-OCT), and for having performed all the scans at the same time in the morning, in order to avoid diurnal variations of choroidal thickness, as already reported in literature [2].

However, we would like to point out some methodological concerns on the findings in this study. Firstly, the authors did not specify if the complete eye examination included slit-lamp biomicroscopy, color fundus photography, axial length and anterior chamber measurements, ophthalmoscopy fundus examination and the measurement of intraocular pressure, central corneal thickness and ocular perfusion pressure (OPP), all parameters that have been shown to affect the choroidal thickness [3]. In this regard, the Beijing Eye Study showed a significant positive association between a thicker choroid and ocular biometric parameters such as a shorter axial length, a deeper anterior chamber depth and a flatter cornea [4]. Moreover, Kim et al. [5] demonstrated that choroid thickness was significantly associated with OPP in healthy subjects. Thus, given the strong effect displayed by the above-mentioned parameters on choroidal thickness, a more detailed disclosure of the ophthalmological examination should have been provided.

Secondly, in this study the authors found a thicker choroid in pregnant women than non- pregnant women, however the results were not statistically significant (304.1 + 9.6 µm in the non-pregnant group vs 318.1 + 15.6 µm in the pregnant group, *p* = 0.446); these findings could have been limited by the relatively small sample size (27 healthy pregnant women in the examined group and 34 healthy non-pregnant women in the control group) [1]. In fact, Liu et al. [6] reported a in a meta-analysis evaluating 8 clinical studies a significantly greater choroidal thickness in pregnant women compared with non-pregnant women with the same age. The underlying mechanism behind the increase of choroidal thickness would reside in the haemodynamic and hormonal changes occurring in pregnancy, with an increased blood flow in this tissue combined with a decreased vascular resistance. In fact, the choroid is a tissue rich of vessels, providing the 85% of ocular perfusion and therefore it is the first ocular layer to be affected by the overall increase of blood volume during pregnancy [7].

In conclusion, further larger scale studies with broader sample sizes should be performed, in order to better describe the complex mechanism of choroidal thickness changes during pregnancy.

## Abbreviations

EDI-OCT: enhanced depth imaging optical coherence tomography
OCT: optical coherence tomography
OPP: ocular perfusion pressure
SD-OCT: spectral-domain optical coherence tomography
